# Effect of forceful suction and air disinfection machines on aerosol removal

**DOI:** 10.1186/s12903-023-03369-1

**Published:** 2023-09-08

**Authors:** Yaru Du, Fei Zhao, Ran Tao, Bing Liu

**Affiliations:** 1 Department of hospital allergy, Medical department, Hebei Key Laboratory of Stomatology, Hebei Clinical Research Center for Oral Diseases, School and Hospital of Stomatology, Hebei Medical University, Shijiazhuang, 050017 PR China; 2 Department of Periodontal I, Hebei Key Laboratory of Stomatology, Hebei Clinical Research Center for Oral Diseases, School and Hospital of Stomatology, Hebei Medical University, Shijiazhuang, 050017 PR China; 3 Medical department, Hebei Key Laboratory of Stomatology, Hebei Clinical Research Center for Oral Diseases, School and Hospital of Stomatology, Hebei Medical University, Shijiazhuang, 050017 PR China

**Keywords:** Air disinfection machines, Forceful suction, PM

## Abstract

**Backgrounds:**

Dental procedures involving drilling and grinding can produce a significant amount of suspended aerosol particles (PM) and bioaerosols. This study aims to analyze the size and concentration of aerosol particles generated during drilling and to investigate the effectiveness of two air exchange systems, namely forceful suction (FS) and air disinfection machines (DM), in removing PM.

**Methods:**

For this study, 100 extracted permanent teeth were collected and divided into three groups: without suction (n = 50), suction with forceful suction (n = 25), and suction with air disinfection machines (n = 25). The removal rate of suspended aerosol particles was analyzed using particle counters and air data multimeter.

**Results:**

When drilling and grinding were performed without vacuum, 0.75% of the aerosol particles generated were PM2.5-10, 78.25% of total suspended aerosol particles (TSP) were PM2.5, and 98.68% of TSP were PM1. The nanoanalyzer measurements revealed that the aerodynamic diameter of most aerosol particles was below 60 nm, with an average particle diameter of 52.61 nm and an average concentration of 2.6*10^11^ ultrafine aerosol particles. The air change per hour (ACH) was significantly lower in the air disinfection machines group compared to the forceful suction group. Additionally, the number of aerosol particles and mass concentration was significantly lower in the air disinfection machines group compared to the forceful suction group in terms of PM2.5 levels. However, the forceful suction group also reduced the mass concentration in PM10 level than the air disinfection machines group.

**Conclusion:**

In conclusion, the air exchange system can reduce the aerosol particles generated during drilling and grinding. Comparing the two air exchange systems, it was found that the air disinfection machines group reduces the number of aerosol particles and mass concentration in PM2.5 levels, while the forceful suction group reduces the mass concentration in PM10 level.

**Supplementary Information:**

The online version contains supplementary material available at 10.1186/s12903-023-03369-1.

## Introduction

Dental caries and periodontal disease are prevalent conditions that pose a significant threat to oral health [1, 2]. Common therapeutic interventions for these diseases encompass restorative dentistry, extractions, scaling, and endodontic treatments [3, 4]. Within the context of dental procedures, aerosols primarily emanate from oral fluids produced by patients, consequently leading to the dissemination of microorganisms and compromising the environmental quality of dental offices [5]. High-speed dental equipment utilization or rinsing during scaling primarily engenders the generation of these aerosols [6, 7], wherein various bacteria, such as Streptococcus spp., have been found in dental offices [8]. Importantly, even upon completion of treatment, noteworthy concentrations of airborne bacteria persist within the dental office vicinity, with potential for dissemination to other treatment areas [9]. Consequently, this scenario poses potential health risks for both patients and dental practitioners [10].

Furthermore, as a supplement to standard protective measures like wearing masks and gloves during dental treatments, the utilization of appropriate preoperative mouth rinses and high-volume rinses is recommended to mitigate the risk of infection [6, 11]. Despite implementing standard safeguards for dentists and staff during dental procedures, exposure to aerosols and particles remains a possibility [12]. Previous studies have suggested a plausible association between aerosol exposure and an elevated risk of respiratory, liver, kidney, and neurological dysfunction [13]. Nevertheless, there exists a dearth of information regarding the relationship between dentist health and aerosol exposure during dental treatments.

revious research has demonstrated a noteworthy increase in the production of particulate matter (PM) by dentists during surgical procedures [14]. Similarly, clinical studies conducted in China have also observed significantly elevated PM levels in the dental working environment, in comparison to non-working states [15]. In order to mitigate aerosol contamination within the dental setting, several clinical measures can be implemented. Firstly, regular disinfection of the dental office environment and equipment is crucial [16]. Secondly, the installation of valves and filters on treatment chairs serves to prevent the negative impact of liquids, aerosols, and PM when utilizing suction devices [17]. However, it is important to note that the efficacy of these measures may be limited, potentially leaving dental professionals and patients vulnerable to the inadvertent transmission of various factors that lack evident symptoms [18]. Furthermore, due to the inability of patients to wear masks during treatment, the development of effective aerosol prevention measures within dental offices presents a significant challenge [19]. Therefore, it is imperative to explore more effective approaches to reduce the presence of bacteria, aerosols, and PM.

Mechanical ventilation and air filtration are regarded as preferable preventive measures for mitigating the risk of airborne diseases within the dental office environment. The Centers for Disease Control and Prevention (CDC) recommends enhancing ventilation systems and incorporating air purifiers in dental offices, thereby minimizing potential risks associated with aerosols [20]. Nonetheless, until recent times, limited attention has been given to the size and concentration of PM produced during dental treatments. Consequently, the objective of this study is to assess the efficacy of two types of air removal devices, specifically forceful suction and air disinfection machines, for the purpose of removing bacteria, PM, and aerosols from the dental office environment.

## Materials and methods

### Tooth sample collection

The collection of tooth samples for this study was conducted in accordance with the ethical standards set forth by the ethics committee of Hebei Medical University and the institutional review board (IRB: 2,011,322,332). Prior to sample collection, all patients were fully informed of the study’s purpose and procedures and provided written informed consent. The study adhered to the guidelines outlined in the CONSORT statement and was conducted in compliance with the Declaration of Helsinki. Inclusion criteria for the study required poor prognosis and the extraction of teeth under constant pressure. A total of 100 teeth were collected from recruited patients, which were obtained from specimens acquired by professional dentists treating severe periodontal disease or severe caries. All samples had fully or partially filled occlusal surfaces and were rapidly extracted and placed in sterile distilled water.

### Experimental model for particle exposure assessment

To simulate a dentist operating in an office setting, we utilized a head shape model based on a previous study [5]. The mobile dental equipment(Zhuhai Duojun Biotechnology Co., Ltd. Zhuhai, China) controlled the drilling and grinding of dental samples, while the high-speed rotating instrument had a speed and efficiency similar to clinical use and produced 50–60 LPM of water to reduce the temperature of the drilling surface [5]. The rotary speed and work efficiency of the high-speed instrument was 350,000–400,000 cycles per minute and 50–60 LPM of water was produced to reduce the temperature of the drilling surface.

### The efficiency of forceful suction and air disinfection

The pump delivery system was adjusted to a flow rate of 8.5 L per minute (LPM) to replicate the nasal inhalation of a human wearing a surgical mask. The mask was secured tightly to the face using taped fittings along the edges to ensure a perfect fit. Two air removal systems were employed to evaluate the efficiency of PM removal.

Prior to commencing drilling or grinding, the indoor background particle concentration was measured. The grinding process was performed for a duration of 2 min, and air samples were collected using a central vacuum system. Each of the 100 teeth was drilled using a new grinding drill. The size and concentration of aerosol particles were measured at a distance of 15 cm from the tooth. Of the 100 teeth, 50 were treated with a N95 mask (Winner Cor. Guanzhou, China) only, 25 with a N95 mask (Winner Cor. Guanzhou, China) and forceful suction (Philips Cor. Netherlands), and 25 with a N95 mask (Winner Cor. Guanzhou, China) and air disinfection machines(Philips Cor. Netherlands).

Particle counters (model 1.109, Grimm Labortechnik Ltd., Ainring, Germany) were employed to assess the size of aerosol particles generated during the drilling and grinding of teeth. The detector was capable of detecting aerosol particles ranging from 0.26 to 34 mm and recorded data every 6 s. The concentrations of PM ≥ 0.5 (aerodynamic diameter ≥ 0.5 μm), PM10 (≤ 10 μm), PM2.5 (≤ 2.5 μm), PM2.5–10 (2.5 < da ≤ 10 μm), and PM1 (≤ 1 μm) were recorded. Hand-held nanoanalyzers (NanoFCM Co., Ltd, Lincoln,USA) were used to detect aerosol particles ranging from 10 to 300 nm and to detect changes in nanoparticle concentration.

### Room airflow and mechanical ventilation rates

The study measured the volumetric airflow rates of enclosed dental treatment rooms and open bay clinics in cubic feet per minute (CFM or ft3/min), using an air velocity sensor integrated into a flow hood (ADM-850 L Airdata Multimeter with CFM-850 L FlowHood, Shortridge Instruments, Inc., Scottsdale, AZ). For metric unit conversion, 1 CFM equals 0.0283 cubic meters per minute (m3/min). The manufacturer calibrated the flow hood of Airdata multimeter following a program that complies with the ANSI/NCSL Z540-1, ISO 17,025, and MIL-STD 45,662 A standards immediately before the experiments. The volumetric sizes of the dental treatment rooms and open bay clinics were calculated in cubic feet (CF or ft3), based on the length, width, and ceiling height of each space. The mechanical ventilation rates of each space in the number of air changes per hour (ACH) were calculated as in previous studies [21, 22].

### Statistical analyses

Statistical analyses were conducted by repeating each experiment five times. SPSS 22.0 software (SPSS, Chicago, Illinois) was used for statistical analyses, with a significance level set at 0.05. GraphPad Prism 6.0 software (GraphPad Software, Inc., USA) was used to graph data. The Wilcoxon signed-rank test was used to determine the removal efficiency of aerosol particles of various sizes (PM ≥ 0.5, PM10, PM2.5, PM1) with or without central vacuum during molar grinding. The Mann-Whitney U test was used to compare differences in the PM levels of drilling teeth and indoor air background, as well as in the filtration efficiency of forceful suction and air disinfection machines.

## Results

In this study, we conducted an analysis of the concentration of total particulate matter (PM) before and after teeth grinding. 50 cases in no suction group and 50 cases in suction group. In addition, for suction group, 25 people in the forceful suction group and 25 people in the air disinfection group.

**Fig. 1:**
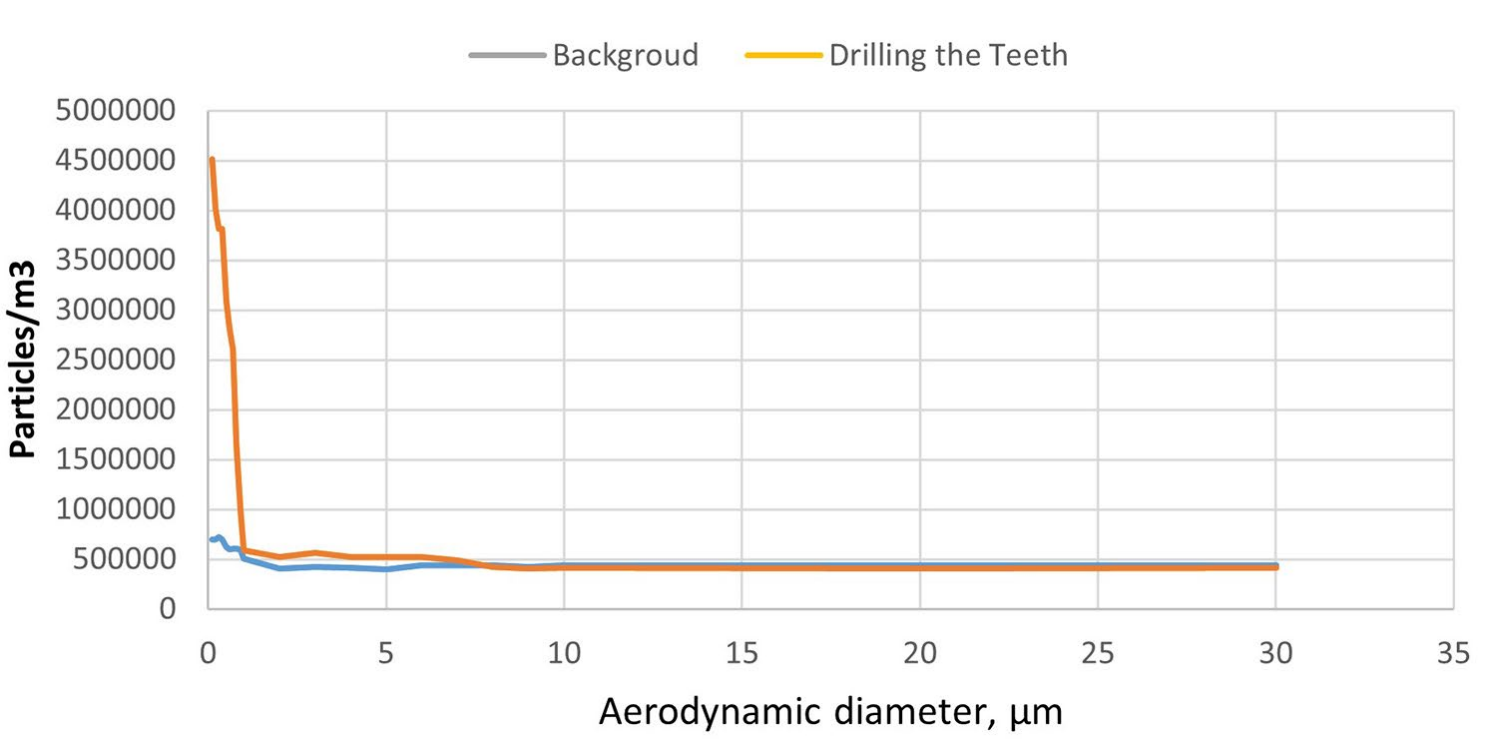
Particle size distributions of drilling the teeth and non-drilling the teeth in non-suction conditions

**Fig. 2:**
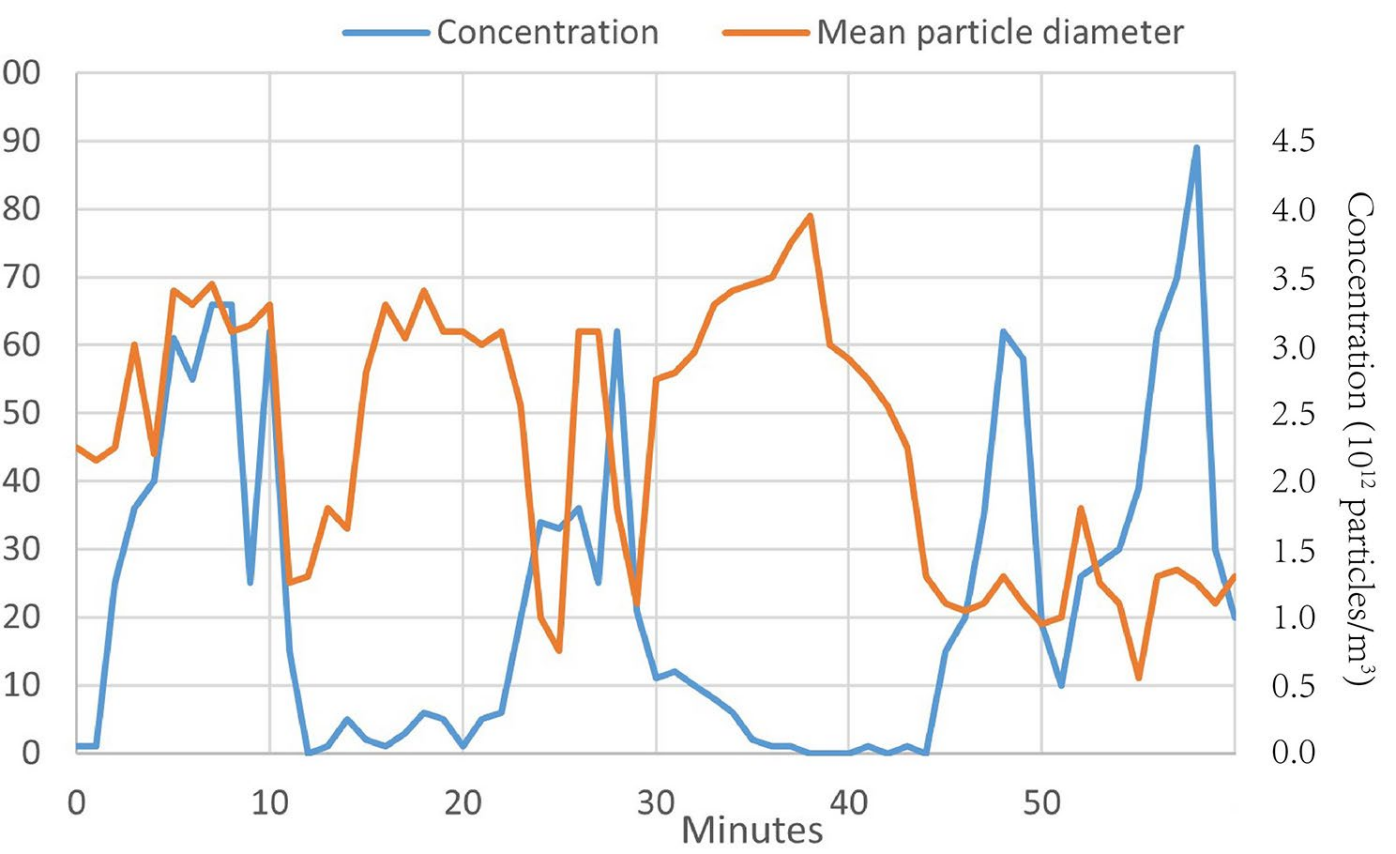
Particle size and concentration distributions of ultrafine aerosol particles generated from drilling teeth procedures

Our findings indicate that the concentration of total PM generated during teeth grinding was significantly higher than the indoor background concentration (p < 0.001, as shown in Fig. 1). When using the drilling and grinding process without vacuum, 0.75% of the aerosol particles in the drilling and grinding process were PM2.5-10, 78.25% of TSP were PM2.5, and 98.68% of TSP were PM1. Furthermore, the nanoanalyzer measurements revealed that the majority of aerosol particles had an aerodynamic diameter below 60 nm, with an average particle diameter of 52.61 nm and an average concentration of 2.6*10^11^ ultrafine aerosol particles (as shown in Fig. 2).

We also investigated the impact of air suction devices on airborne aerosol particles. Our results demonstrate that the air changes per hour (ACH) level significantly decreased in the suction devices group (p < 0.001, as shown in Table 1). Additionally, the number concentration of PM ≥ 0.5 and PM2.5 was significantly lower in the suction group compared to the group without suction. Mass concentrations of PM such as PM ≥ 0.5, PM10, PM1.0, and PM2.5 were also significantly lower in the suction group compared to the group without suction (as shown in Table 1).

**Table 1:** Comparison of particle concentrations under drilling the teeth without/with suction conditions

		Without suction (n = 50)	With suction (n = 50)	P value
Volume ft3(m3)		956.69(63.25)	597(42.18)	0.025
ACH		16.25 ± 1.59	8.62 ± 2.06	< 0.001
Temp(℃)		24.61 ± 3.62	20.62 ± 1.89	0.562
RH(%)		44.28 ± 2.61	41.25 ± 3.69	0.251
Number concentration, particles/m3	PM ≥ 0.5	1.88*10^7^(1.06*10^7^-2.65*10^7^)	8.88*10^6^(5.6*10^6^-9.95*10^6^)	0.011
	PM10	1.52*10^5^(1.22*10^5^-1.71*10^6^)	8.39*10^4^(2.6*10^4^-8.5*10^4^)	0.035
	PM2.5	1.06*10^5^(1.84*10^4^-6.65*10^5^)	8.01*10^4^(3.8*10^4^-9.2*10^4^)	0.011
	PM1	1.38*10^5^(0.76*10^5^-1.77*10^5^)	7.58*10^4^(4.9*10^4^-1.02*10^5^)	< 0.001
Mass concentration, µg/m3	PM ≥ 0.5	135.66(72.61-196.25)	69.25(43.61–99.25)	< 0.001
	PM10	81.05(60.22-102.28)	29.68(10.02–45.62)	< 0.001
	PM2.5	19.05(11.06–29.36)	9.05(2.98–11.25)	0.002
	PM1	6.00(2.61–9.05)	2.66(0.99–3.98)	< 0.001

Furthermore, we analyzed the effects of different air exchange machine, such as forceful suction and air disinfection machines, during teeth grinding. Our analysis revealed that the ACH was significantly lower in the air disinfection machines group compared to the forceful suction group. Additionally, the number and mass concentration of aerosol particles were significantly lower in the air disinfection machines group compared to the forceful suction group in terms of PM2.5 levels. However, the forceful suction group also reduced the mass concentration in PM10 level compared to the air disinfection machines group (p = 0.011, supplement Table 1).

**Fig. 3:**
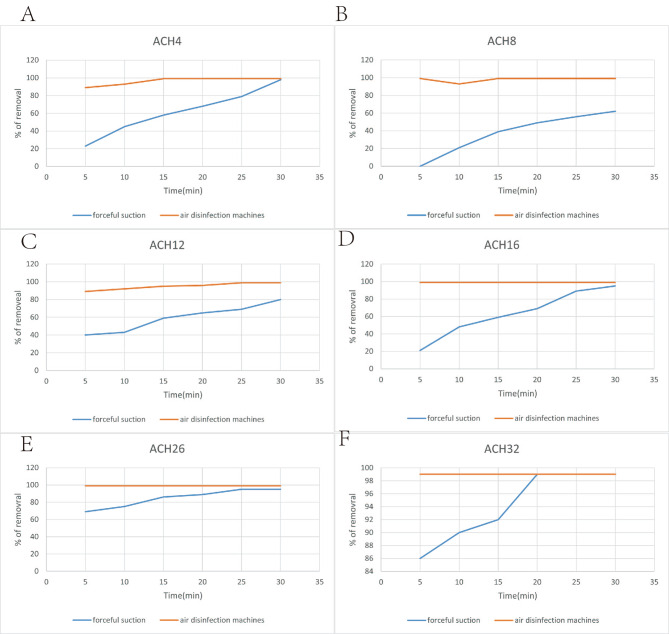
Removal efficiency for the 0.3 μm aerosol aerosol particles with forceful suction and air disinfection machines at 4, 8, 12, 16, 26 and 32 min after aerosol generations in dental treatment rooms with various mechanical ventilation rates measured by air change per hour (ACH).

**Fig. 4:**
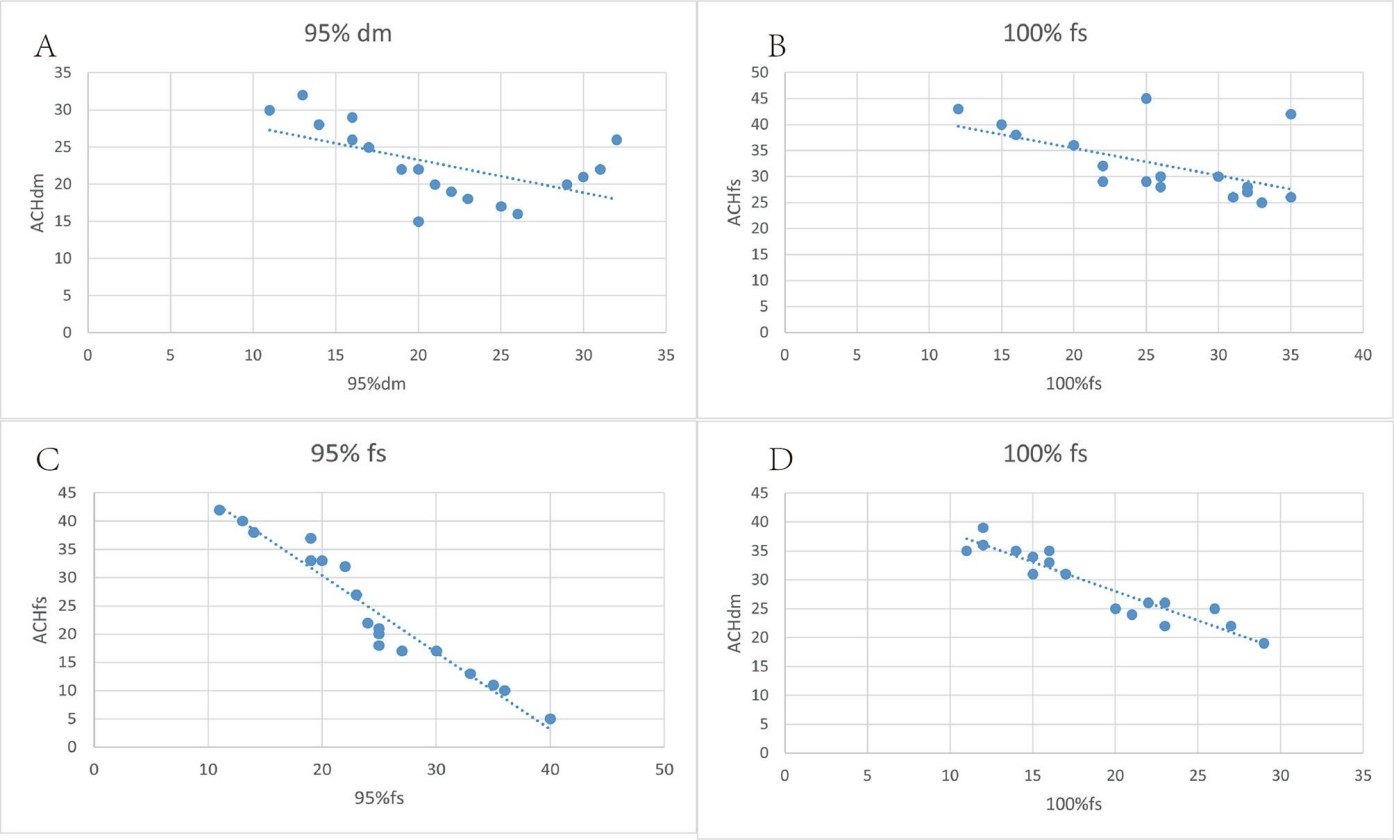
Correlations between air disinfection machines (ACHdm) or forceful suction (ACHfs) and times needed to 95% or 100% removals of the 0.3 μm aerosol aerosol particles. Higher ACHdm(A, B) and ACHfs(C,D) are highly correlated with shorter times needed to reach 95% or 100% aerosol removal

Figure 3 demonstrates that air disinfection machines (DM) were more effective in removing aerosol particles than forceful suction (FS) at 4, 8, 12, 16, 26, and 32 min. Our analysis of different air removal devices revealed that ventilation systems were significantly associated with air disinfection machines, and we correlated the time taken for air changes per hour (ACH) with 95% and 100% aerosol particle removal. The correlation coefficients were high between ACHdm and the time needed to reach 95% (r=-0.92, p < 0.01) and 100% (r=-0.85, p < 0.01) aerosol particle removals with air disinfection machines. Similarly, the correlation was clear between ACH forceful suction and the time needed to reach 95% (r=-0.89, p < 0.05) and 100% (r=-0.75, p < 0.05) aerosol removals with the forceful suction group, as illustrated in Fig. 4.

## Discussion

In the discussion, it is noted that the drilling and grinding of teeth can increase the concentration of suspended aerosol particles in the dental environment. However, other sources can also contribute to higher particle concentrations in dental offices [23]. A recent study in the United States found that indoor aerosol particles in dental offices were primarily composed of aerosol particles 6500 nm in size, with ultrafine aerosol particles (< 100 nm in size) accounting for 67% of all aerosol particles [24]. Our study also found a significant increase in aerosol particles after the start of drilling, and these fine aerosol particles may penetrate deep into the lungs through the alveoli of the respiratory system [25].

Dental droplets or aerosols, generated by the aerodynamic forces resulting from high-speed rotations or ultrasonic vibrations of dental instruments, possess the potential to project into the air within dental treatment rooms, leading to contamination of the indoor environment [11, 26]. The differentiation between droplets and aerosols has traditionally been established based on their physical size, with particles larger than 5 μm in diameter being classified as droplets that exhibit rapid descent to the ground and limited travel within a 2-meter radius. In contrast, particles smaller than 5 μm are referred to as droplet nuclei or aerosols that can remain suspended in the air over an extended duration, traveling distances greater than 2 m [27]. Dental droplets or aerosols, propelled by the aerodynamic forces of high-speed rotation or ultrasonic vibration from dental instruments, can disseminate into the air space of the dental treatment room, leading to contamination of the indoor environment [11, 26]. The distinction between droplets and aerosols is primarily attributed to variations in particle size, where droplets larger than 5 μm in diameter rapidly descend to the ground, covering distances no greater than 2 m. In contrast, droplet nuclei and aerosols smaller than 5 μm can persist in the air for prolonged periods [28]. The smaller the aerosol particles, the longer their airborne suspension, consequently increasing the risk of inhalation by individuals in the vicinity. Although low- and high-volume vacuum evacuation serve as effective measures for controlling droplets and aerosols, their implementation can reduce microbial and particle contamination by more than 90%. However, it is important to acknowledge that the health implications associated with these minute aerosol particles within the dental environment still require further investigation. Nevertheless, the Centers for Disease Control and Prevention (CDC) advises dental professionals to restrict aerosol-generating procedures and implement measures to enhance ventilation and air filtration measures during the ongoing COVID-19 pandemic [29]. S Given the prolonged suspension of aerosol particles within this size range in the air, improving ventilation and utilizing air filtration systems emerge as the most feasible and efficacious approaches to eliminate them from the indoor environment [30, 31]. Aerosol refers to a dispersion system comprising solid or liquid aerosol particles suspended in a gaseous medium, which can be generated by people, animals, instruments, or machines. In December 2019, an outbreak of pneumonia caused by a novel coronavirus infection emerged in China, with respiratory droplet transmission and close contact transmission being the primary modes of transmission [32–34]. In relatively closed environments with prolonged exposure to high concentrations of aerosols, the possibility of aerosol transmission cannot be ruled out [35–37]. Given that dental treatments generate a large number of droplets and aerosols carrying pathogenic microorganisms, there is a high risk of in-hospital spread and hospital infection [38–40].

Furthermore, comprehending the transmission of respiratory pathogens holds paramount importance for implementing effective public health measures aimed at mitigating their spread [41, 42]. The generation and size of particles play pivotal roles in the carriage, atomization, and dissemination of pathogens. The generation of infectious respiratory particles hinges upon the type and frequency of respiratory activities, the nature and site of infection, and the pathogen load [43]. Additionally, relative humidity, particle aggregation, and mucus properties exert influence over the size of expelled particles and their subsequent dispersion [44].

The dental profession possesses unique characteristics, whereby the examination, diagnosis, and treatment procedures are conducted within the confines of the dental office. Within this integrated treatment space, various factors such as doctor-patient communication and patient behavior (e.g., coughing, sneezing, gargling) can generate droplets. Additionally, the use of high-speed ultrasonic equipment during treatment can produce a substantial amount of aerosols and droplets [45–48]. Droplets that are less than 5 μm in diameter remain suspended in the air, and the nucleus of droplets formed after dehydration can move with the air, thus leading to environmental pollution in the treatment area [44, 49]. Larger diameter droplets tend to settle quickly in the vicinity of the droplet source. If not processed in a timely manner, the settled droplets can form droplet nuclei after drying, which can be mechanically suspended into the air again, resulting in secondary pollution [50, 51]. Therefore, it can be inferred that the primary components of the aerosol in the dental treatment area are the aerosol generated by the treatment process, the droplet nucleus formed after drying the droplet sprayed into the air, and the droplet nucleus raised again after drying and dehydration of the larger diameter droplet settlement.

Oral treatment is a continuous and dynamic process, where the number of chairs in operation during a given period of time directly affects the bacterial content in the air and the settling time required after the treatment. A study revealed that two hours after the ultrasonic scaling operation, the bacterial content in the air increased by 4.8 times in a five-chair office and 4.3 times in a single-chair office. Furthermore, one hour after the treatment, the bacterial colonies in the air decreased by 45% in a five-chair office, which was 2.3 times more than before the opening, and 39% in a single-chair office, which was 1.9 times more than before the opening [52, 53]. The range of bioaerosol contamination varies depending on the operation, with aerosols from ultrasonic scaling spreading up to 1 m horizontally and 0.5 m vertically, and spattering distances up to 1.6 m horizontally and 1.8 m vertically for dental preparation. In a closed room, aerosol contamination is more extensive and can reach the entire room [54–56].

There is a clear association between fixed particles and pulmonary complications. First of all, there is a clear correlation between the time solid particles stay in the lungs, and the larger the particles, the longer the lung remains [57]. Second, there is also a clear association between solid particles and the incidence of lung disease [58]. One of the most notable instances of an air pollution catastrophe occurred in London in December 1952, resulting in an estimated count of over 4,000 deaths [59]. Air pollution comprises a intricate amalgamation of gases and particles. Gaseous pollutants infiltrate deeply into the alveoli, facilitating their diffusion across the blood-air barrier to affect numerous organs [60]. On the other hand, particulate matter (PM) encompasses a concoction of solid or liquid particles suspended in the atmosphere. Depending on their size, coarse particles (PM10) settle in the upper airways, whereas fine particles (PM2.5) can accumulate within the lung parenchyma, instigating various respiratory ailments. Besides size, the composition of PM has been linked to diverse toxicological consequences based on clinical, epidemiological, in vivo, and in vitro animal and human studies. PM can consist of organic, inorganic, and biological compounds, all of which possess the capability to alter several biological activities, including cytokine production, coagulation factor equilibrium, pulmonary function, respiratory symptoms, and cardiac function [61, 62]. As it traverses the airways, the exposure to air pollution can engender various alterations, including the recruitment of inflammatory cells and the subsequent release of cytokines and reactive oxygen species (ROS). These inflammatory agents have the capability to activate distinct signaling pathways, such as MAP kinases, NF-κB, and Stat-1, as well as induce DNA adducts [63]. Collectively, these modifications can contribute to the development of obstructive or restrictive pulmonary diseases, encompassing conditions such as asthma, chronic obstructive pulmonary disease (COPD), pulmonary fibrosis, and even cancer [64]. In 2013, based on a comprehensive analysis of research studies pertaining to the effects of air pollution, the International Agency for Research on Cancer (IARC) classified outdoor air pollution as Group 1 [65]. Harrel SK et al. found that aerosol concentrations were high within a 2-foot (0.609 m) range centered on the patient [11, 66]. Zhang Yuqin et al. demonstrated that the degree of contamination in the dental office was inversely proportional to the distance from the treatment site, decreasing with increasing distance [67]. Our study employed two air exchange systems that significantly improved airborne aerosol particles, not only in terms of concentration but also in terms of particle size. This finding is consistent with previous studies that suggest suction devices may reduce aerosol concentrations during dental procedures [68]. Additionally, our results indicated that the air disinfection machines group significantly reduced airborne aerosol particles compared to the forceful suction group.

Limitations of this study include the challenge of accurately quantifying the precise exposure of dental staff. Although we utilized a simulation system to analyze the removal of airborne aerosol particles, it may not fully reflect actual working conditions. Furthermore, other factors such as sound, humidity, and wind speed are crucial in determining particle distribution and require further investigation in future studies.

## Conclusion

In conclusion, the air exchange system can effectively decrease the number of aerosol particles generated during dental procedures involving drilling and grinding. Comparing the two air exchange systems, we found that the group utilizing air disinfection machines demonstrated a more significant reduction in suspended aerosol particles compared to the forceful suction group.

### Electronic supplementary material

Below is the link to the electronic supplementary material.

Supplementary Material 1

## Data Availability

All data generated or analysed during this study are included in this published article.

## Abbreviations

PM: Suspended particles
FS: Forceful suction
DM: Air disinfection machines
TSP: Total suspended particles
CDC: Centers for Disease Control
LPM: Liter per minute
ACH: Air change per hour
ACHdm: Air disinfection machines
ACHfs: Forceful suction
