# Correction to: Effect of forceful suction and air disinfection machines on aerosol removal

**DOI:** 10.1186/s12903-023-03774-6

**Published:** 2023-12-19

**Authors:** Yaru Du, Fei Zhao, Ran Tao, Bing Liu

**Affiliations:** 1 Department of infection Management, medical department, Hebei Key Laboratory of Stomatology, Hebei Clinical Research Center for Oral Diseases, School and Hospital of Stomatology, Hebei Medical University, Shijiazhuang, 050017 PR China; 2 Department of Periodontal I, Hebei Key Laboratory of Stomatology, Hebei Clinical Research Center for Oral Diseases, School and Hospital of Stomatology, Hebei Medical University, Shijiazhuang, 050017 PR China; 3 Medical department, Hebei Key Laboratory of Stomatology, Hebei Clinical Research Center for Oral Diseases, School and Hospital of Stomatology, Hebei Medical University, Shijiazhuang, 050017 PR China

**Correction to: BMC Oral Health (2023) 23:652**

10.1186/s12903-023-03369-1

In this article [1], the first author affiliation needs to be revised as “Department of infection Management, medical department, Hebei Key Laboratory of Stomatology, Hebei Clinical Research Center for Oral Diseases, School and Hospital of Stomatology, Hebei Medical University, Shijiazhuang, 050017, PR China”.
