# Supplementary material for: Effect of forceful suction and air disinfection machines on aerosol removal
Source: BMC Oral Health. 2023 Sep 8;23:652. doi: 10.1186/s12903-023-03369-1 (PMC10492290; doi:10.1186/s12903-023-03369-1)
Supplement: Supplementary file 1 — Supplementary Material 1 [file 12903_2023_3369_MOESM1_ESM.docx]

| Supplement Table 1. Comparison of particle concentrations under drilling the forceful suction and Air disinfection machines. | | | | |
| --- | --- | --- | --- | --- |
|  | | forceful suction (n = 25) | Air disinfection machines (n = 25) | P value |
| Volume ft3 (m3) | | 693.64±52.98 | 412.06±48.99 | 0.032 |
| ACH | | 16.25±1.59 | 5.99±0.97 | 0.048 |
| Temp(℃) | | 24.61±3.62 | 14.02±4.02 | 0.115 |
| RH(%) | | 44.28±2.61 | 23.94±6.85 | 0.026 |
| Number concentration, particles/m3 | PM≥0.5 | 1.99 | 0.82 | 0.158 |
|  | PM10 | 1.82 | 0.81 | 0.112 |
|  | PM2.5 | 2.11 | 0.58 | 0.015 |
|  | PM1 | 1.58 | 0.51 | 0.147 |
| Mass concentration, μg/m3 | PM≥0.5 | 1.62 | 1.11 | 0.129 |
|  | PM10 | 1.05 | 1.56 | 0.011 |
|  | PM2.5 | 1.52 | 1.18 | 0.032 |
|  | PM1 | 1.26 | 1.09 | 0.015 |
